# The Effect of Laminin Surface Modification of Electrospun Silica Nanofiber Substrate on Neuronal Tissue Engineering

**DOI:** 10.3390/nano8030165

**Published:** 2018-03-14

**Authors:** Wen Shuo Chen, Ling Yu Guo, Chia Chun Tang, Cheng Kang Tsai, Hui Hua Huang, Ting Yu Chin, Mong-Lin Yang, Yui Whei Chen-Yang

**Affiliations:** 1 Center for Nanotechnology, Center for Biomedical Technology, Department of Chemistry, Chung Yuan Christian University, Taoyuan 32023, Taiwan; allanson92@yahoo.com.tw (W.S.C.); sunny_day80917@yahoo.com.tw (L.Y.G.); wesley741129@gmail.com (C.C.T.); chengkang20071994@gmail.com (C.K.T.); jenny16155@gmail.com (H.H.H.); 2 Department of Bioscience Technology, Chung Yuan Christian University, Taoyuan 32023, Taiwan; tychin@cycu.edu.tw; 3 Department of Science, Concordia University Saint Paul, Saint Paul, MN 55104, USA

**Keywords:** electrospun silica nanofibers, surface modification, laminin, synergistic effect, neuronal tissue engineering

## Abstract

In this study, we first synthesized a slow-degrading silica nanofiber (SNF2) through an electrospun solution with an optimized tetraethyl orthosilicate (TEOS) to polyvinyl pyrrolidone (PVP) ratio. Then, laminin-modified SNF2, namely SNF2-AP-S-L, was obtained through a series of chemical reactions to attach the extracellular matrix protein, laminin, to its surface. The SNF2-AP-S-L substrate was characterized by a combination of scanning electron microscopy (SEM), Fourier transform–infrared (FTIR) spectroscopy, nitrogen adsorption/desorption isotherms, and contact angle measurements. The results of further functional assays show that this substrate is a biocompatible, bioactive and biodegradable scaffold with good structural integrity that persisted beyond 18 days. Moreover, a synergistic effect of sustained structure support and prolonged biochemical stimulation for cell differentiation on SNF2-AP-S-L was found when neuron-like PC12 cells were seeded onto its surface. Specifically, neurite extensions on the covalently modified SNF2-AP-S-L were significantly longer than those observed on unmodified SNF and SNF subjected to physical adsorption of laminin. Together, these results indicate that the SNF2-AP-S-L substrate prepared in this study is a promising 3D biocompatible substrate capable of sustaining longer neuronal growth for tissue-engineering applications.

## 1. Introduction

Great advances in nanotechnology have recently propelled the development of numerous biomaterials utilizing natural, synthetic, or hybrid composites for applications in the biomedical sciences and engineering fields [1,2,3,4]. Specifically, recent studies have shown how various nanoscale biomaterials can be applied to great effect in areas such as cancer therapy [5,6], gene delivery [7,8], antimicrobial resistance [9,10] and tissue engineering [11,12,13,14]. In this study, we seek to optimize the ability of a nanoscale silica fiber to serve as a substrate for neuronal tissue engineering.

With the world’s fast-growing population, nerve damage caused by increasing incidents of automobile accidents, sports injuries, and war wounds, etc. are rapidly growing as well. To assist in injury healing, tissue-function restoration and nervous-system regeneration, biomaterial scaffolds for tissue engineering have been extensively developed and implemented [15,16,17]. Most recent studies on neuronal tissue engineering have focused on producing scaffolds, which mimic the extracellular matrix (ECM), including being three-dimensional, bioactive, and biodegradable [18]. Many of these scaffolds are either natural or synthetic organic polymers, with some that are commercially available for human peripheral nerve regeneration [15,16,17]. However, many of these scaffolds exhibit various disadvantages which limit their application, including the inconsistency from batch to batch of natural materials, and the required use of toxic organic solvents for the dissolution of synthetic organic polymers [19]. Therefore, novel nontoxic and biocompatible inorganic-based materials have also been examined and explored as a desirable alternative [20]. Among several methods for the preparation of ECM-mimicking scaffold materials [12,21], electrospinning is a convenient technique typically used in the preparation of nanofibers for in vitro experiments [20,22,23,24,25,26,27,28,29,30]. However, if materials need to be mass produced for clinical use, other possible methods such as pressure-coupled infusion gyration [31] can be considered. Our previous studies utilizing electrospun silica nanofibers (SNF) as a neuronal tissue-engineering material have shown that it is a three-dimensional, bioactive and biodegradable inorganic scaffold material [23,24]. Specifically, the biodegradability of the electrospun SNF (SNF1) required no enzyme treatment and could simply degrade in a short time frame [24]. While this short time frame is potentially ideal for early-release drug delivery applications, it is likely faster than desired for tissue-engineering applications, where persistent mechanical support is necessary for healthy neuronal cell growth. Neuronal cells, unlike other somatic cells, have unique morphological extensions that require lengthened mechanical support as well as continuous chemical-signal stimulation [32,33,34]. In this study, we set out to prepare and characterize an improved version of our SNF scaffold that can address these issues and provide a more robust neuronal tissue-engineering scaffold.

Recent literature emphasizes the importance of providing surface contact guidance cues for developing neurons in the form of ECM proteins. For example, a synthetic organically-based polycaprolactone (PCL) nanofibrous scaffold was unable to direct neural stem-cell differentiation towards neurons for the regeneration of large gaps in peripheral nerve tissue when lacking ECM proteins as contact guidance cues [35]. Laminin, a component of the ECM, has been shown to serve as an ideal biochemical cue for promoting and sustaining the differentiation of neuronal cells [35,36]. Many studies have incorporated laminin onto their scaffolds of choice and have observed enhanced neurite outgrowth. However, most of these studies investigated cell growth and differentiation only up to 10 days at most in vitro. For example, the proliferation of Schwann cells on the laminin-modified PCL-chitosan nanofiber substrate was tracked for 4 days [25], and on a laminin-modified PLCL core-shell nanofiber substrate for 7 days [26]. The neurite extensions of neuron-like PC12 cells on laminin-modified PLLA nanofiber substrates were observed for 5 days [27], and on a covalently-bonded laminin-PCL nanofiber for 10 days [28]. The neurite extension of DRG on a laminin-PCL blend nanofiber was traced for 4 days [29] and on a laminin-modified PLLA nanofiber for 6 days [30]. In addition, the spontaneous induction of neurite extensions of PC12 cells on PGS-PMMA/Gel50 nanofibers were observed for 8 days [14]. Taken together, these studies indicate that a combination of the electrospun nanofiber structure and bioactive cues provides a potentially ideal nerve scaffold in short-term in vitro cell culture. Nevertheless, it is important to remember that longer-term 3D culture which can both mechanically and chemically sustain differentiation is required for neuronal tissue regeneration. In fact, some studies have observed that degradation of the scaffold structure or erosion of surface modification over longer incubation periods has a significant effect on cell growth. For example, a previous study has shown that when alginate was not cross-linked into hydrogel, it was not suitable for long-term culture [37], and the scaffolds attached with the ECM protein fibrin extended the degradation from 7 days to 30 days, leading to a study of neural stem-cell differentiation for 14 days [38]. Given this evidence, we first sought to alter our electrospinning solution recipe to enhance cross-linkage within the nanofibers. We hypothesized that more extensive cross-linkage through a higher degree of condensation in a sol-gel reaction would delay the rate of scaffold degradation, thereby extending the scaffold’s physical support for neurite growth and extension. Second, we hypothesized that introducing covalently-bonded laminin to our scaffold surface would provide more robust and persistent biochemical cues, prolonging the stimulation of neurite extension.

To find an ideal cell model for the assessment of longer-term neuronal differentiation on our SNF scaffold, we looked to the neuron-like rat pheochromocytoma cell line, PC12. In previous studies [23], we found that neural stem/progenitor cells quickly overgrow and cover the surface of the substrate within 5 days, limiting our ability to track cell differentiation and material dissolution for the longer-term. Moreover, PC12 cells attach poorly to uncoated glass and serve as a more sensitive model than primary neurons to test the erosion of biochemical cues from the surface of the scaffolds as well as material cell interactions. Thus, in this study, we set out to prepare a laminin surface-functionalized SNF (SNF2-AP-S-L) that is: (1) slow-degrading, providing enhanced physical support for neurite outgrowth, and (2) biochemically sustaining, to allow prolonged chemical stimulation that promotes neurite extension. We assessed SNF2-AP-S-L through long-term in vitro growth/differentiation of PC12 cells and found it to sustain neurite extension beyond 18 days, with no observed retraction and continual lengthening.

## 2. Materials and Methods 

### 2.1. Materials

Tetraethyl orthosilicate (TEOS) and (3-aminopropyl) trimethoxysilane (APTS) was purchased from Acros Organics (Thermo Fisher Scientific, Waltham, MA, USA). Polyvinyl pyrrolidone (PVP, Mw = 1,300,000 g/mol), phosphate-buffered saline (PBS), Laminin and 4-(N-maleimidomethyl) cyclohexane-1-carboxylic acid 3-sulfo-N-hydroxysuccinimide ester sodium salt (sulfo-SMCC) were purchased from Sigma-Aldrich Co. (St. Louis, MO, USA).

### 2.2. Preparation, Modification, and Characterization of Silica Nanofiber (SNF) Substrates

The silica nanofibers, SNF2, were prepared by an electrospinning method according to the procedures reported previously [23,24]. Briefly, 1.9 g of the silica precursor, TEOS, 3.15 g of ethanol, 2.0 g of water and 0.04 g of formic acid were mixed with 0.9 g of PVP. The mixture was continuously stirred for 1 h to form a homogeneous solution, in which at least 300 cP of viscosity is required for the production of the continuous fiber mat. The solution was ejected at a flow rate of 0.9 mL/h through a 24-G plastic syringe with a stainless steel needle at a distance of 10 cm from a 12 × 12 mm^2^ coverslip placed on a flat aluminum plate collector under a voltage of 16 kV to produce the silica/PVP composite fibers. The silica/PVP composite fibers collected on the coverslip were then subjected to calcination for 3 h at 450 °C to remove the PVP and the solvent residues to obtain SNF2. 

**Figure 1:**
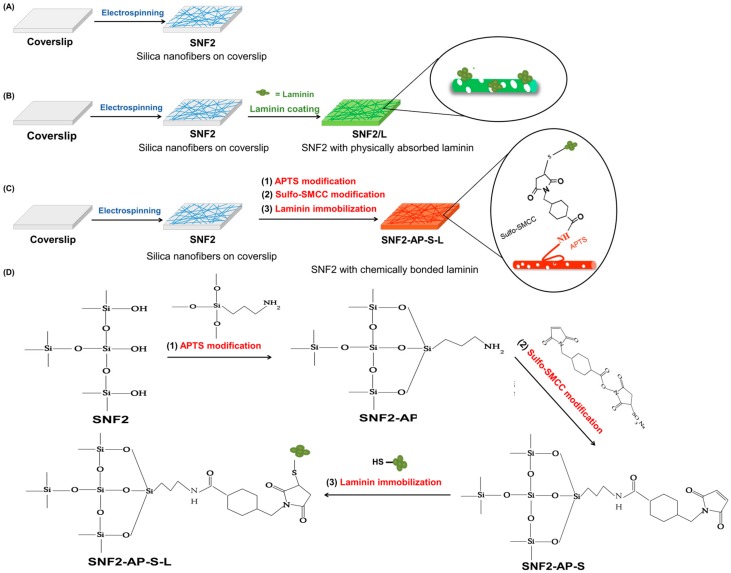
Schematic of the surface modifications of the three SNF2 substrates. (**A**) Silica nanofibers (SNF2) electrospun onto coverslip; (**B**) physically laminin-adsorbed SNF2 (SNF2/L) (**C**) chemically laminin-bonded SNF2 (SNF2-AP-S-L); (**D**) the chemical reactions for the preparation of SNF2-AP-S-L.

For functionalization of the surface, the as-prepared SNF2 was modified by laminin with two methods, namely the physical coating method and the chemical bonding method as shown in Figure 1. For the physical coating method, the as-prepared SNF2 substrate was immersed in a 0.01 mg/mL of laminin solution for 24 h at 4 °C, then rinsed with sterile 0.01 M PBS for three times to obtain the laminin-coated SNF (denoted as SNF/L). For the chemical bonding method, the SNF2 substrate was immersed in an ethanol solution with 3 M APTS for 24 h at room temperature and washed with PBS to obtain the APTS-modified SNF2 (SNF2-AP) as described before [23,24]. Then, the solution of the cross-linker, sulfo-SMCC, in PBS was added in SNF2-AP for 2 h at room temperature [39] to obtain the APTS-SMCC-modified SNF2 (SNF2-AP-S). Finally, the SNF2-AP-S nanofibers were rinsed three times with sterile 0.01 M PBS and immersed in a 0.01 mg/mL of laminin solution (in PBS) for 24 h at 4 °C and dried completely to obtain the chemical bonded laminin-functionalized SNF2 (denoted as SNF2-AP-S-L) and kept sterile.

The morphologies of all the as-prepared SNFs and the modified substrates were investigated with a S-3500N field emission scanning electron microscope (SEM) (Hitachi, Tokyo, Japan). The diameters of the as-prepared SNFs (50 fibers for each sample, *n* = 50) were measured from the SEM images using Image J analysis software. Also, Fourier transform infrared (FTIR, BIORad-FTS-7, Perkin Elmer, Waltham, MA, USA) spectra of SNF2, SNF2-AP, SNF2/L, and SNF2-AP-S-L substrates were measured over 4000–400 cm^−1^ at a resolution of 2 cm^−1^ to characterize the functional groups of the SNFs prepared. To determine the specific surface area of the as-prepared SNFs, the nitrogen adsorption/desorption isotherms were measured using a Tristar 3000 analyzer (Micromeritics, Norcross, GA, USA) and analyzed with the Brunauer–Emmett–Teller (BET) method. Water contact angles of the as-prepared SNF substrates were measured using an FTA 125 analyzer (First Ten Angstroms, Portsmouth, VA, USA) at ambient temperature. Specifically, 4 μL water droplets were carefully and separately dropped onto different positions of the sample surface. The average value of the contact angles of all the droplets (at least 15 droplets, *n* = 15) measured from 3 different samples was calculated. The silica structures of the nanofibers were measured by the ^29^Si magic angle spinning nuclear magnetic resonance spectrometer (MAS NMR, Bruker DSX400WB, Billerica, MA, USA).

### 2.3. In Vitro Culture of PC12 Cells

Rat PC12 cells (American Type Culture Collection, Manassas, VA, USA) were used to study the effect of the laminin-modified SNFs, SNF-AP-S-L and SNF/L, on neuronal differentiation. PC12 cells have previously been used to explore biomaterials for nerve regeneration [24,40]. PC12 cells were cultured in high-glucose Dulbecco’s modified Eagle’s medium (DMEM) supplemented with 5% heat-inactivated fetal bovine serum, 10% horse serum and 1% penicillin/streptomycin at 37 °C in a humid atmosphere with 5% CO_2_ according to the study previously described [41]. The live cells were counted using trypan blue exclusion assay in a hemocytometer. SNF substrates on the coverslips were placed under the UV radiation overnight before use. For neuronal differentiation, cells were seeded and grown at a density of 6.9 × 10^3^ cells/cm^2^ onto the desired surfaces of substrates and cultured in DMEM supplemented with 1% heat-inactivated fetal bovine serum, 2% horse serum and 1% penicillin/streptomycin supplemented with 100 ng/mL NGF (Corning, Corning, NY, USA). The medium was replenished every 3 days.

### 2.4. Silica Degradation Study

The degradation experiments were conducted to evaluate the effect of the degree of crosslinking on the degradation of the as-prepared SNF substrates. The SNF substrates were incubated in a 12-well plate at 37 °C in PBS or DMEM with daily exchange for an extended period. After 6 days, 12 days and 18 days in 0.010 M PBS, fiber morphology was observed with SEM. After 12 days and 18 days in DMEM, the distribution of the laminin coating on the as-prepared substrates was examined by the immunocytochemistry method with rabbit anti-laminin primary antibody [42]. First, the substrates for non-specific labeling were blocked with 10% goat serum (Thermo Fisher Scientific). The samples were then immersed in the rabbit anti-laminin primary antibody (diluted at 1:500, Sigma). After that, the samples were incubated with DyLight 488 conjugated donkey anti-rabbit (1:250 dilution; Jackson ImmunoResearch, West Grove, PA, USA) antibody for 1.5 h at 37 °C, and ten images at different positions were viewed under fluorescence microscopy (Nikon, Shinagawa, Tokyo, Japan).

### 2.5. Cell Viability

PC12 cell viability was determined using a LIVE/DEAD assay kit (Life Technologies, Waltham, MA, USA), which contained Calcein AM and EthD-1 dyes at a concentration of 1 μM. For the determination, PC12 cells were seeded on the as-prepared SNF substrates then assayed after 72 h cultivation, according to the procedure reported previously [24,42]. The dyes diluted in PBS were added to the cultivated cells for 10 mins then 12 images at different positions (*n* = 12) were taken for each sample utilizing the fluorescence microscope (Nikon). The neurons stained with the cell-permeant dye, Calcein AM, were counted as live cells, whereas those stained with the membrane-impermeable DNA-binding dye, EthD-1, were counted as dead cells. The percentage of viable cells was calculated based on the ratio of the number of live cells to that of the total cells for each sample.

### 2.6. Immunocytochemistry Staining

Fluorescence immunocytochemistry was conducted for the PC12 cells according to the procedure reported [43,44]. The cells were fixed on the substrate with 4% paraformaldehyde (PFA, Merck & Co. Inc., Kenilworth, NJ, USA) for 30 min and blocked with 10% goat serum and 0.3% Triton X-100 in PBS for 2 h. The cells were then incubated overnight at 4 °C with rabbit anti-microtubule-associated protein 2 (MAP2) antibody (1:500 dilution; Chemicon). Furthermore, the same cells were incubated with secondary antibodies, DyLight 488 conjugated donkey anti-rabbit (1:250 dilution; Jackson ImmunoResearch) antibody for 1.5 h at 37 °C. The fluorescence images of various fields of the cells that resulted were acquired using fluorescence microscopy (Nikon). Thirty images were recorded for each sample at 20× objective lens and the neurite lengths were analyzed with the Neuron J software [45] plugin of Image J [46] to trace the neurite for length measurement.

### 2.7. Laminin Erosion Fluorescence Analysis

Fluorescence images of the as-prepared SNF substrates from different days of degradation study were first obtained as described in Section 2.4. For each sample, utilizing the ImageJ software, 10 pairs of regions of interest (ROI), including those from fibers and those from the corresponding backgrounds, were randomly selected. The average signal intensity of each region was measured by tracing 50-pixel line either along the fiber or the background. The ratio of the average signal intensity of ROI obtained from the fiber over that from the corresponding background was then calculated and reported.

### 2.8. Cell Morphology Study

The morphology of the PC 12 cells on the as-prepared SNF substrates, were observed with SEM. On day 6, day 12, and day 18, the substrates with cells were collected, fixed with formaldehyde and sequentially dehydrated through an increasing concentration gradient of alcohol. Finally, substrates were dried at room temperature and coated with gold for observation with the SEM.

### 2.9. Statistical Analysis

All the data presented are expressed as means ± standard error of mean. The difference was determined by Student’s *t*-test or one-way analysis of variance (ANOVA). Statistical significance was taken at *p* < 0.05.

## 3. Results and Discussion

### 3.1. Preparation and Characterization of SNF2

Silica nanofibers (collectively SNF2) were synthesized using an electrospinning solution containing a higher ratio of silica precursor TEOS vs. PVP than the formulation previously used in preparation of SNF1 [23]. We hypothesized that this formulation change would increase the degree of condensation, resulting in (1) a higher degree of cross-linkage within formed fibers, and (2) increased fiber diameter, two qualities that would retard fiber-degradation rate. The degree of condensation was determined by solid-state ^29^Si-NMR spectroscopy, in which spectral chemical shifts represent the environment of the ^29^Si atoms. In general, the chemical shift (relative to tetramethylsilane) at about −80, −90, −100 and −110 ppm are designated to Q^1^, Q^2^, Q^3^ and Q^4^ for Si(OSi)(OH)_3_, Si(OSi)_2_(OH)_2_, Si(OSi)_3_OH and Si(OSi)_4_), respectively. The broad peak in the SNF2 spectrum was deconvoluted into three peaks (Q4 (−110 ppm), Q3(−101 ppm), and Q2 (−92 ppm)) to determine the degree of condensation, using an algorithm reported previously [47]. Table 1 shows that the degree of condensation was higher for SNF2 (91.8%,) than that previously reported for SNF1 (76.2%) [23], validating our hypothesis regarding reactivity of the electrospinning solution. In addition, SEM imaging analysis revealed that fiber diameter increased from an average length of 260 ± 68 nm in SNF1 to an average length of 620 ± 79 nm in SNF2 (*n* = 50), reflecting a similar result reported previously [48]. Last, SNF2 was found to have a slightly smaller surface area (309 m^2^/g versus 366 m^2^/g) and pore size (2.1 nm versus 3.4 nm) compared to SNF1.

### 3.2. Degradation of SNF2 in Physiological Buffer

**Figure 2:**
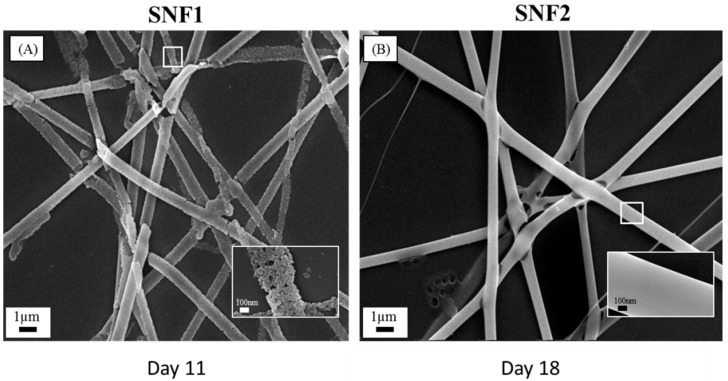
Scanning electron microscope (SEM) images of (**A**) SNF1 immersed in 10 mM PBS with daily buffer exchange at 37 °C after 11 days and (**B**) SNF2 immersed in 10 mM phosphate-buffered saline (PBS) with daily buffer exchange at 37 °C after 18 days. The insets are the magnification of the corresponding surfaces. The scale bars represent 1 μm in (**A**,**B**) and 100 nm for the insets.

Having verified that our SNF2 substrate exhibits a greater degree of cross-linkage and increased fiber diameter, we quantified its degradation rate relative to SNF1. Both SNF1 and SNF2 were immersed in PBS with daily buffer exchange; SEM imaging reveals that SNF2 was able to maintain its structural integrity beyond 18th days (Figure 2). Using higher magnification, we observed that the fiber surface of SNF2 at day 18 maintained better structural integrity than that of SNF1 at day 11 (Figure 2). Thus, SNF2 exhibits significantly delayed degradation compared to SNF1, which had barely any structure left by the 15th day [24].

### 3.3. Preparation and Characterization of Laminin Modified SNF2, SNF2/L, and SNF2-AP-S-Ls

Various studies on synthetic neuronal tissue engineering scaffolds have proven the benefit of laminin modification in promoting and sustaining neurite growth [27,29,49]. We hypothesized that modification of SNF2 surfaces with laminin would not only serve to promote cell adhesion but would also provide a biochemical cue to sustain neurite outgrowth. In addition, we hypothesized that covalent bonding of laminin onto SNF2 would prevent erosion of laminin over time, thereby supporting long-term neurite extension. To test the above hypotheses, we prepared three substrates for analysis. As shown in Figure 1, we prepared three substrates for analysis: SNF2 without additional modification (Figure 1A); SNF2/L, a preparation of SNF2 physically absorbed with laminin (Figure 1B); and SNF2-AP-S-L, where SNF2 was chemically modified and subsequently reacted with laminin, resulting in covalent thiol-maleimide linkage of laminin to SNF2 (Figure 1C).

The physical adsorption of laminin onto the SNF2 surface was performed via a simple incubation of 0.01 mg/mL laminin solution with SNF2 substrate for 24 h. Covalent linkage of laminin onto the SNF2 surface was accomplished by surface-grafting of APTS to SNF2 (generating SNF2-AP), followed by reaction with SMCC (generating SNF2-AP-S), and finally reaction with laminin, resulting in covalent crosslinking between the maleimide of SMCC and the thiol moeties of cysteine residues within laminin (Figure 1D). 

**Figure 3:**
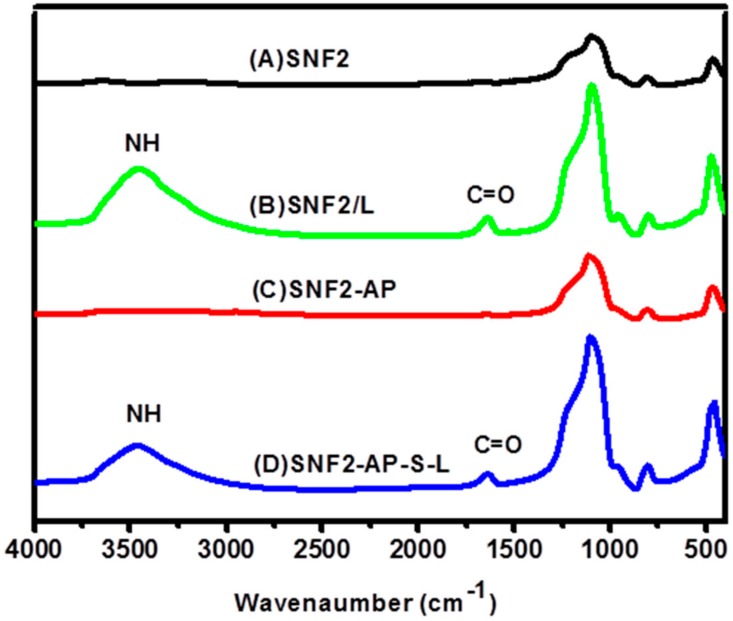
Fourier transform–infrared (FTIR) spectra of (**A**) SNF2, (**B**) SNF2/L, (**C**) SNF2-AP and (**D**) SNF2-AP-S-L.

FTIR analysis was employed to validate the successful physical adsorption of laminin onto SNF2 surface to create SNF2/L. As seen in Figure 3B, the characteristic bands of peptide bonds (1700–1600 cm^−1^ for C=O stretching vibration of amide I; 3500–3300 cm^−1^ for N-H stretching vibration) [50] indicate that the peptide bond of laminin was successfully introduced on the surface of physically adsorbed silica nanofibers (SNF2/L). This result is consistent with earlier studies [51,52].

To ensure the successful covalent linkage of laminin onto SNF2, SNF2-AP-S-L, we first examined the grafting of APTS onto the SNF2 surface using FTIR. Figure 3C shows the characteristic bands of ATPS in the SNF2-AP FTIR spectra (2970 cm^−1^ for –CH; 3500–3300 cm^−1^ of –NH stretching vibration; 1650–1400 cm^−1^ of –NH bending vibration). After crosslinking of laminin onto SNF2-AP, FTIR analysis again shows the characteristic peptide bonds of SNF2-AP-S-L as shown in Figure 3D.

**Figure 4:**
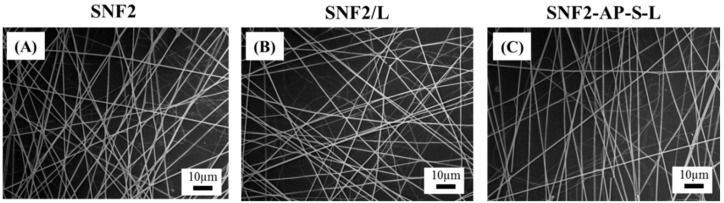
SEM images of (**A**) SNF2, (**B**) SNF2/L and (**C**) SNF2-AP-S-L. Scale bar represents 10 μm.

The physical properties of the three substrates were quantified and compared using SEM imaging, contact angle measurement, and BET isotherm analysis. As shown in Table 2 and Figure 4, the SEM micrographs showed that the fiber diameters of SNF2 (620 ± 77.8 nm), SNF/L (636 ± 62 nm) and SNF-AP-S-L (636 ± 62 nm) substrates did not change significantly (*n* = 50, one-way ANOVA, *p* > 0.1). In addition, a typical SNF2 substrate had a thickness of about 74 μm as shown in Figure S1. Water contact-angle measurements indicate that physical modification of laminin slightly increases the hydrophobicity of the surface from 42.1 ± 4.9° in SNF2 to 50.5 ± 1.3° in SNF2/L (*n* = 15). For the covalently modified substrate, the surface hydrophobicity increased to 102 ± 1.8° after APTS attachment, and decreased to 54.7 ± 10.5° after laminin crosslinking (*n* = 15), comparable to SNF2/L. BET isotherm analysis indicated that laminin-modified substrates had a significantly smaller surface area, and significantly larger pore size, than unmodified SNF2. One likely explanation for this observed difference is the filling of smaller pores by laminin in the modified substrates, resulting in a reduction of surface area and overall pore volume while increasing the measured average pore size. This paradoxical observation has also been discussed in our previous publication as potential proof of successful surface modification [24].

### 3.4. Degradation of SNF2/L and SNF2-AP-S-L in Physiological Buffer

**Figure 5:**
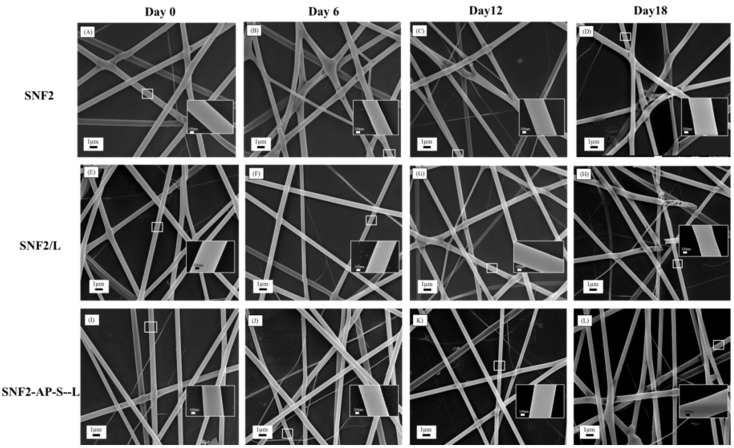
SEM images of SNF2 (**A**–**D**), SNF2/L (**E**–**H**), and SNF2-AP-S-L (**I**–**L**) after being immersed in 10 mM PBS with daily buffer exchange for different time periods at 37 °C. (**A**,**E**,**I**) at Day 0, (**B**,**F**,**J**) at Day 6, (**C**,**G**,**K**) at Day 12, (**D**,**H**,**L**) at Day 18. Scale bars represent 1 μm in (**A**–**L**) and 100 nm for the insets.

Evaluation of SNF2/L and SNF2-AP-S-L degradation in PBS buffer at 37 °C with daily buffer exchange was performed as before, with SEM images taken at three different time points: 6, 12, and 18 days. Figure 5D,H,L show that fibers of all three samples retained their fiber integrity through 18 days with only minor breakages on the 18th day. Higher magnification of the fibers (inset) shows no significant change in surface morphology. In fact, in addition to uniformly smooth surface appearance, observed cross sections of broken fibers indicated robust structural integrity. This result reaffirms that fibers made using the SNF2 formulation are more stable substrates potentially suitable for longer-term neuronal differentiation.

### 3.5. SNF2-AP-S-L as a Longer-Term Biocompatible Substrate That Promotes Neurite Extension

Having demonstrated the desired improvements to substrate physical properties, we next employed PC12 cells in biological testing of our three SNF2 substrates. Unlike the neuronal stem cells used previously, the culturing of differentiated PC12 cells avoids the problem of rapid proliferation leading to overpopulation of the substrate, which prevents long-term observation. Moreover, compared to primary neurons, PC12 cells attach poorly to the uncoated glass surface [53], thus making them a more sensitive reporter of laminin erosion.

**Figure 6:**
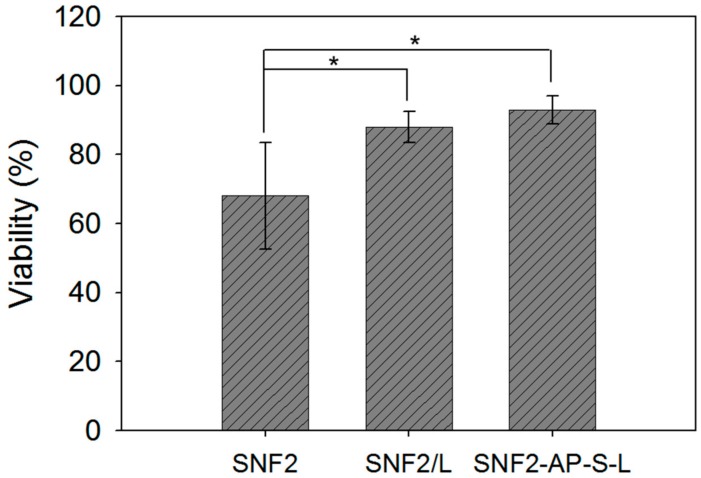
Cell viability comparison between PC12 cells cultured on the as-prepared SNF2, SNF2/L, and SNF2-AP-S-L substrates. Assessments were done using LIVE/DEAD^®^ stain with Ethidium Homodimer-1 and Calcein AM 72 h after seeding. *n* = 12 *t*-test, * *p* < 0.05, compared to SNF2.

Next, LIVE/DEAD assays were carried out to establish the biocompatibility of the different surfaces. Figure 6 shows that after 72 h the two laminin-modified surfaces supported better cell viability than the unmodified SNF2. Specifically, reported viability on both SNF2/L (88 ± 4%) and SNF2-AP-S-L (95 ± 2.0%) was significantly higher than unmodified SNF2 (68.3 ± 12.8%) (*n* = 12 *t*-test, *p* < 0.05), indicating that PC12 cells could not easily adhere on the surface of SNF2 without laminin modification. This result reaffirms the biocompatibility of the SNF substrate and demonstrates the importance of laminin coating for sustaining cell growth.

**Figure 7:**
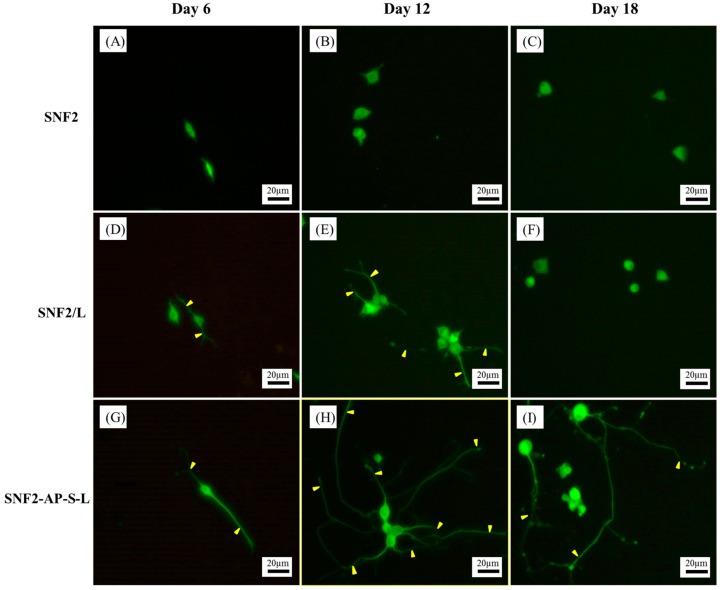
Fluorescence microscopy image of PC12 cells differentiation on SNF2 (**A**–**C**), SNF2/L (**D**–**F**) and SNF2-AP-S-L (**G**–**I**) for different periods of time. Neurites (indicated with arrowheads) were immunostained with MAP2 (green). (**A**,**D**,**G**) at Day 6. (**B**,**E**,**H**) at Day 12. (**C**,**F**,**I**) at Day 18. Scale bars represent 20 μm.

To verify that surface modification with laminin can indeed promotes neurite outgrowth as indicated by previous studies [27], NGF-induced PC12 cell differentiation on SNF2, SNF2/L and SNF2-AP-S-L substrates was assessed via immunocytochemically staining against MAP2. Figure 7 shows fluorescent microscopy images of PC12 neurite outgrowth on the three substrates. The images show that unmodified SNF2 provided negligible support for neurite outgrowth (Figure 7A–C). On the other hand, SNF2/L supported neurite extension until the 12th day (Figure 7E), with significant neurite retraction detected by the 18th day (Figure 7F). As for the chemically modified SNF2-AP-S-L, the images show that this substrate can sustain and promote neurite extension throughout the 18 days observed (Figure 7G–I). 

**Figure 8:**
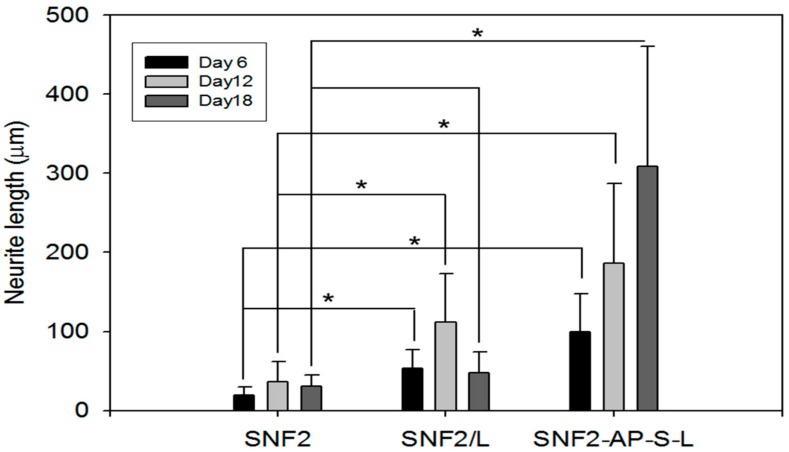
Bar graph comparing the neurite length of PC12 cells cultured on the as-prepared SNF2, SNF2/L and SNF2-AP-S-L substrates at the different time points of Day 6, Day 12 and Day 18 (*n* = 30, *t*-test, * *p* < 0.05).

Neurite length was determined via image analysis software, using methods previously established [45]. As summarized in Figure 8, cells seeded on SNF2 exhibited minimal neurite extension throughout the time observed. Cells on physically-adsorbed SNF2/L exhibited a better extension than SNF2 throughout the 18 days. However, these cells failed to sustain their neurite length from day 12 to day 18 with the average detected neurite length significantly decreasing from day 12 (111.67 ± 61.65 μm) to day 18 (47.64 ± 26.87 μm). Cells on chemically-modified SNF2-AP-S-L exhibited continually-increasing neurite lengths across all time point, from 99.58 ± 47.74 μm on day 6, 186.39 ± 100.45 μm on day 12 to 308.86 ± 151.10 μm on day 18, thus, SNF2-AP-S-L was the only substrate among those surveyed that supported neurite elongation after 18th day without retraction. In summary, these data show that surface modification with laminin does indeed promote neurite outgrowth and extension as previously published [27]. In addition, with extended tracking, we were able to observe that this neurite-promoting ability is sustained over much longer development periods by substrates with chemically-modified surfaces, compared to physically-adsorbed substrates. This result indicates that SNF2-AP-S-L can sustain neurite outgrowth longer than SNF2/L due to the covalent crosslinking of laminin onto SNF2.

**Figure 9:**
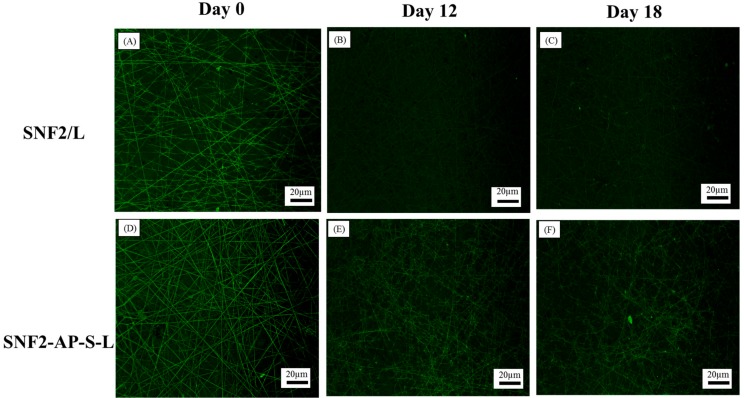
Fluorescence images of SNF2/L (**A**–**C**) and SNF2-AP-S-L (**D**–**F**) substrates immunostained with anti-laminin antibody at different time point: (**A**,**D**) Day 0, (**B**,**E**) at Day 12, (**C**,**F**) at Day 18. Scale bars represent 20 μm.

To compare the rate of laminin erosion on SNF2-AP-S-L and SNF2/L under culturing conditions, we immersed each substrate in culture media with daily exchange for various incubation periods, and subsequently immunostained against laminin. Fluorescent microscopy images shown in Figure 9 illustrate that under the same exposure time, both SNF2-AP-S-L and SNF2/L started at 0 days with a strong fluorescent signal from freshly-coated laminin (Figure 9A,D). However, the signal from SNF2/L shows significant attenuation with time, and by the 18th day the signal was close to the background (Figure 9C). In contrast, while the signal from SNF2-AP-S-L also fades with time, it remains significantly strong by the 18th day, when it appeared to be stronger and more visible than SNF2/L (Figure 9F). 

**Figure 10:**
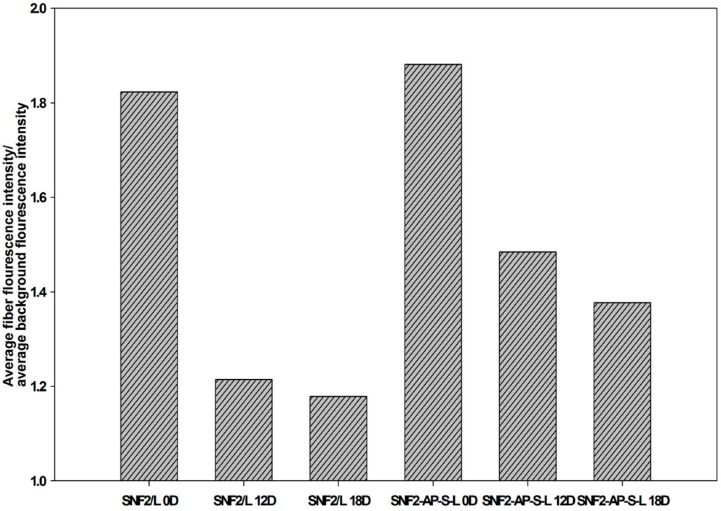
Laminin erosion fluorescence analysis of SNF2/L and SNF2-AP-S-L substrates immunostained with anti-laminin antibody at Day 0, Day 12 and Day 18 (*n* = 10).

To quantify this data, we used the image analysis software, Image J [46] to obtain the average signal intensity from 10 randomly-selected regions of 50 pixels on both the electrospun fiber and the background. The ratio of the average intensities gathered from the fiber over background is reported on the *y*-axis of Figure 10. This data indicate that physically-adsorbed laminin eroded faster as time went on with daily media exchange. SNF2-AP-S-L, which has covalently-crosslinked laminin, retained much more of its fluorescent signal than SNF2/L after 18 days of daily media exchange. This result supports our hypothesis that SNF2-AP-S-L can sustain longer-term neurite outgrowth due to the slower erosion of covalently-crosslinked laminin.

**Figure 11:**
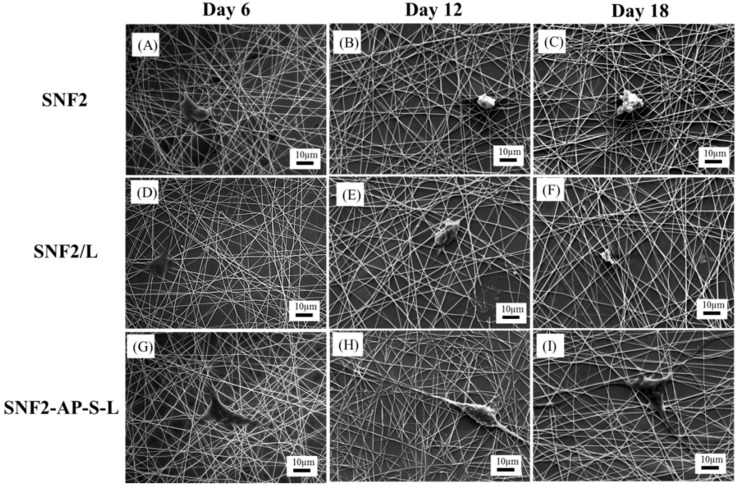
SEM images of PC12 cell differentiation on SNF2 (**A**–**C**), SNF2/L (**D**–**F**) and SNF2-AP-S-L (**G**–**I**) at different time points. (**A**,**D**,**G**) are images taken at Day 6. (**B**,**E**,**H**) are images taken at Day 12. (**C**,**F**,**I**) are images taken at Day 18. Scale bars represent 10 μm.

Last, to visualize the structure and morphology of the cell–scaffold interaction in our substrates, SEM images were analyzed for closer inspection of the cell morphology on all substrates at each time point. SNF2 substrate modified with laminin is found to enhance neurite extension of PC12 cells compared to those on the unmodified SNF2 at 6 days post-differentiation (Figure 11A,D,G). This result is similar to a previous study [27]. Furthermore, SNF2-AP-S-L continued to support cell growth and enhanced the neurite outgrowth of PC12 cells in subsequent days (Figure 11H,I) compared to pure SNF2 (Figure 11B,C) or SNF2/L (Figure 11E,F). The SEM images reaffirm the structural integrity of SNF2 after 18 days in culture and provide a clearer visualization of the cell morphology on the 18th day.

For the SNF2/L with limited laminin remaining on the surface (Figure 11E,F), cells appear round and less defined, implying limited adhesion without differentiation. In contrast, for the SNF2-AP-S-L with ample laminin remaining (Figure 11H,I), cells grown on the surface appeared flatter (indicating cell adhesion) with pronounced protrusions (indicating cell differentiation).

Regarding cell and scaffold interaction Figure 11I shows that the representative PC12 cell on the SNF2-AP-S-L substrate has its cell soma nicely spread out and strongly attached to the substrate surface, and its neurites weaving in and out of the silica nanofibers’ three-dimensional structure. However, the cell on SNF2/L in Figure 11F is retracting and barely hanging on to the surface with much less attachment to the silica nanofiber. This result corroborates the result shown in Figure 8 regarding average neurite length, which indicated retracting of neurite from SNF2/L on day 18. 

Currently, more and more nanotechnology studies are investigating the utilization of inorganic and hybrid substrates as the next-generation materials for gene delivery [7,8], biosensor [54,55], and cancer therapy [5,6]. In the future, we believe that novel biomaterials which combine self-healing hydrogels [56] with robust functionalized nanomaterials will enable great advances for in situ tissue regeneration and engineering in the field of human medicine. The covalently-modified silica nanofiber substrate developed in the present work represents a significant step toward this long-term objective, and future work will continue to explore its potential as a robust neuronal growth substrate for regeneration and tissue-engineering applications.

## 4. Conclusions

In this work, we have successfully carried out the surface functionalization of laminin onto the slower degrading electrospun silica nanofiber, SNF2, through a series of chemical reactions. Our results showed that SNF2 could maintain its structure beyond 18 days, thereby providing more consistent mechanical support for neurite extension. Surface modification through both physical adsorption (SNF2/L) and chemical modification (SNF2-AP-S-L) of laminin promoted neurite growth. However, we found that laminin eroded from SNF2/L at a moderate rate that had significant repercussions on cell growth and differentiation; in contrast, SNF2-AP-S-L was shown to retain surface-modified laminin beyond the 18 days observed, and cells on SNF2-AP-S-L exhibited constant neurite extension. These results reveal a synergistic effect of sustained structural support and prolonged biochemical stimulation for PC12 cells. Therefore, the SNF2-AP-S-L substrate synthesized in this work is a promising, slow-degrading, laminin-functionalized silica nanofiber substrate readily suitable for neuronal tissue engineering.

## Figures and Tables

**Table 1:** Characteristic date of the silica nanofiber, SNF1 and SNF2, substrates.

Sample	Fiber Diameter (nm)	Surface Area (m^2^/g)	Pore Size (nm)	^29^Si NMR Characterization			Cross-Linkage (%)	Reference
				Q^2^	Q^3^	Q^4^		
				(%)	(%)	(%)		
SNF1	260 ± 68	366	3.4	12.4	70.4	17.2	76.2	23
SNF2	620 ± 79	309	2.1	2.1	28.5	69.4	91.8	In this study

**Table 2:** Characterization of the SNF substrates with different surface modification.

Sample	Fiber Diameter (nm)	Contact Angle (°)	Surface Area (m^2^/g)	Pore Volume (cm^3^/g)	Pore Size (nm)
SNF2	620 ± 79	42.1 ± 4.9	309	0.034	2.1
SNF2-AP	643 ± 98.5	102 ± 1.8	14.0	0.027	2.73
SNF2/L	636 ± 62	50.5 ± 1.3	5.9	0.004	11.8
SNF2-AP-S-L	619 ± 85	54.7 ± 10.5	1.2	0.008	51.4

## Supplementary Materials

The following are available online at http://www.mdpi.com/2079-4991/8/3/165/s1, Figure S1: SEM images of SNF2 mat. The scale bar represents 10 μm.
