# Supplementary material for: The Effect of Laminin Surface Modification of Electrospun Silica Nanofiber Substrate on Neuronal Tissue Engineering
Source: Nanomaterials (Basel). 2018 Mar 14;8(3):165. doi: 10.3390/nano8030165 (PMC5869656; doi:10.3390/nano8030165)
Supplement: Supplementary file 1 [file nanomaterials-08-00165-s001.pdf]

Supplementary Information

The Effect of Laminin Surface Modification of Electrospun Silica Nanofiber Substrate on Neuronal Tissue Engineering

Wen Shuo Chen [1], Ling Yu Guo [1], Chia Chun Tang [1], Cheng Kang Tsai [1], Hui Hua Huang [1], Ting Yu Chin [2], Mong-Lin Yang [3,*] and Yui Whei Chen-Yang [1,*]

1 Department of Chemistry, Center for Nanotechnology, Center for Biomedical Technology, Chung Yuan Christian University, Chung Li, Taiwan 32023, Republic of China; allanson92@yahoo.com.tw (W.S.C.); sunny_day80917@yahoo.com.tw (L.Y.G.); wesley741129@gmail.com (C.C.T.); chengkang20071994@gmail.com (C.K.T.); jenny16155@gmail.com (H.H.H.)

2 Department of Bioscience Technology, Chung Yuan Christian University, Chung Li, Taiwan 32023, Republic of China; tychin@cycu.edu.tw (T. Y. C.)

3 Department of Science, Concordia University Saint Paul, Saint Paul, Minnesota, USA.

* Correspondence: myang2@csp.edu (M.-L. Y.); yuiwhei@cycu.edu.tw (Y.W. C-Y.); Tel.: +1-651-6418220 (M.-L.Y.); Tel.: +866-3-2653317 (Y.W. C-Y.)

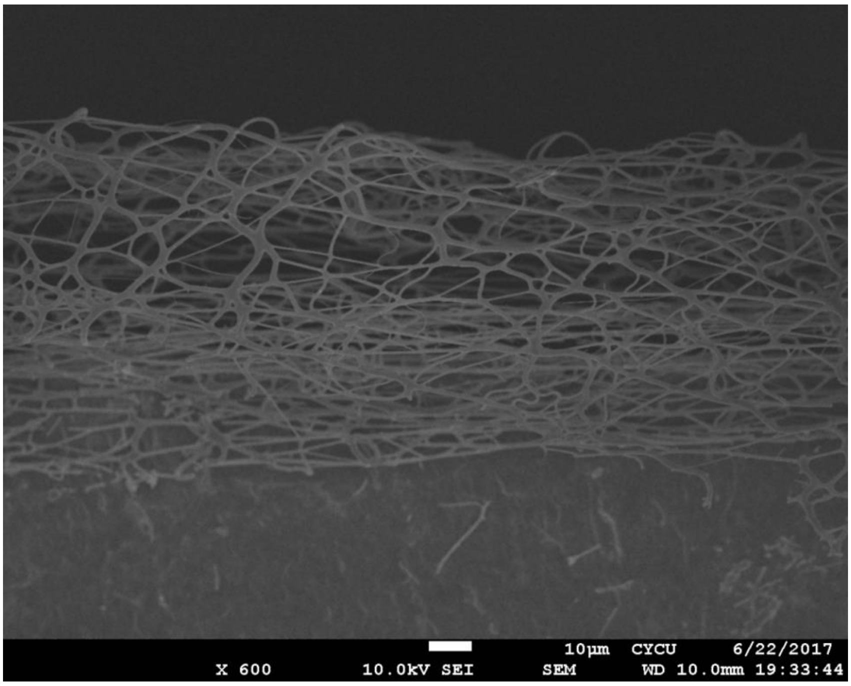

Figure. S1 SEM images of SNF2 mat. The scale bar represents 10 μm.
